# KDM4C (GASC1) lysine demethylase is associated with mitotic chromatin and regulates chromosome segregation during mitosis

**DOI:** 10.1093/nar/gku253

**Published:** 2014-04-11

**Authors:** Ilana Kupershmit, Hanan Khoury-Haddad, Samah W. Awwad, Noga Guttmann-Raviv, Nabieh Ayoub

**Affiliations:** Department of Biology, Technion, Israel Institute of Technology, Haifa 3200003, Israel

## Abstract

Various types of human cancers exhibit amplification or deletion of KDM4A-D members, which selectively demethylate H3K9 and H3K36, thus implicating their activity in promoting carcinogenesis. On this basis, it was hypothesized that dysregulated expression of KDM4A-D family promotes chromosomal instabilities by largely unknown mechanisms. Here, we show that unlike KDM4A-B, KDM4C is associated with chromatin during mitosis. This association is accompanied by a decrease in the mitotic levels of H3K9me3. We also show that the C-terminal region, containing the Tudor domains of KDM4C, is essential for its association with mitotic chromatin. More specifically, we show that R919 residue on the proximal Tudor domain of KDM4C is critical for its association with chromatin during mitosis. Interestingly, we demonstrate that depletion or overexpression of KDM4C, but not KDM4B, leads to over 3-fold increase in the frequency of abnormal mitotic cells showing either misaligned chromosomes at metaphase, anaphase–telophase lagging chromosomes or anaphase–telophase bridges. Furthermore, overexpression of KDM4C demethylase-dead mutant has no detectable effect on mitotic chromosome segregation. Altogether, our findings implicate KDM4C demethylase activity in regulating the fidelity of mitotic chromosome segregation by a yet unknown mechanism.

## INTRODUCTION

Nucleus of the eukaryotic cells is composed of DNA and proteins that are organized in higher-order structure termed chromatin (1). One main function of chromatin is to allow sophisticated packaging of the DNA into the eukaryotic nucleus. The second function is to provide a dynamic platform that regulates the execution of diverse processes such as replication, gene expression, DNA repair and recombination (2–6). Dysregulation of these processes or mutations affecting chromatin-remodeling complexes has been linked to many multi-system disorders and cancer development (3,7,8).

Chromatin structure is regulated by the dynamic interplay between histone-interacting proteins and post-translational modifications (PTMs) of histones (5,9,10). This regulation is capable of altering the chromatin and therefore modulates DNA accessibility (10,11). Histones are subjected to a variety of PTMs such as acetylation, methylation, phosphorylation, ubiquitylation, sumoylation, ADP-ribosylation, deimination and proline isomerization (12). These PTMs have multiple functions including the regulation of gene transcription (2,13). Accordingly, PTMs regulate the establishment and maintenance of transcriptionally active euchromatin and the condensed form of chromatin named heterochromatin (13–17).

Lysine methylation is one of the most common modifications of histone tails, which acts as a platform for chromatin modifier proteins and leads to either gene activation or repression. Tri-methylation of histone H3 lysine 9 (H3K9me3) is enriched in condensed pericentric heterochromatin, while di-methylation of histone H3 lysine 9 (H3K9me2) and mono-methylation of histone H3 lysine 9 (H3K9me1) are associated with transcriptionally silent regions within euchromatin (18–20). Further, lysine methylation has been implicated in multiple cellular processes such as regulation of gene expression, DNA replication, recombination, repair, heterochromatin formation and maintenance, mitosis and genomic imprinting. Aberrant histone methylation has also been linked to different human diseases such as cancer (21–31).

Two families of lysine demethylases (KDM) have been identified, confirming that histone methylation is a reversible and dynamically regulated process (32–34). One family is referred to as the Jumonji C (JmjC)-domain-containing proteins. This class contains the conserved JmjC catalytic domain, which belongs to the cupin superfamily of metalloenzymes (35). The crystal structure of the JmjC catalytic domain was solved and found to form an enzymatically active pocket that coordinates the two co-factors that are needed for the radical-based oxidative demethylation reaction, ferrous oxide (Fe(II)) and α-ketoglutarate (35–37).

The human KDM4 family consists of four members, KDM4A-D (also known as JMJD2A-D). These enzymes specifically catalyze the demethylation of H3K9me2/me3, H3K36me2/me3 and H1.4K26me2/me3 in a Fe^2+^ and α-KG-dependent manner (37). Besides the catalytic JmjC domain, KDM4 demethylases contain the JmjN domain, which is also required for the demethylase activity (38–40). In addition, all KDM4 members, except the shortest KDM4D protein, contain two Plant homeodomain (PHD) and two Tudor domains. It was shown that PHD and Tudor domains are not required for KDM4 enzymatic activity (39,41). On the other hand, it was found that deletion of JmjN, PHD or Tudor domains resulted in both nuclear and cytoplasmic localization of KDM4A and KDM4C proteins, suggesting that these domains are essential for the nuclear localization of KDM4 proteins (39,41). In agreement with this, the Tudor domains of KDM4A isoform bind H4K20me2, H4K20me3, H3K4me3 and H3K9me3, while the PHD domains were found to bind H3K4me3 (41–44). Thus, binding of KDM4A and KDM4C isoforms to chromatin via their Tudor and PHD domains may assist with their localization in the nucleus (41). Notably, the Tudor domain is a conserved protein structural motif, which is also present in many proteins that function in RNA metabolism, histone modification, DNA damage response, development, differentiation and cell division (45–49).

KDM4 proteins are involved in a plethora of cellular processes including gene expression regulation (50–54), DNA replication (21,55), DNA damage response (56–58), worm development and germ cell apoptosis (34), renewal of embryonic stem cells (59), and male lifespan in Drosophila (60). Interestingly, various types of human cancer show misregulated expression of KDM4A-D members (61–63). For example, KDM4C (also known as GASC1) is amplified in esophageal squamous carcinomas, medulloblastomas and breast cancer. The depletion of KDM4C inhibits proliferation of several tumor cell lines, while overexpression of the protein induces transformed phenotypes in mammary epithelial cells (24,50,64–69). Likewise, KDM4B overexpression promotes gastric tumorigenesis and its depletion leads to cell-cycle arrest and apoptosis of gastric cancer cell lines. Additionally, KDM4B depletion causes a decrease in colony formation and cell proliferation of estrogen-receptor-positive breast cancer cells and impairs therefore the development of normal breast tissues *in vivo* (70–72). Altogether, these observations suggest that overactivity of KDM4 proteins have a causative role in tumorigenesis, and therefore understanding the mechanisms by which KDM4 proteins promote chromosomal instability becomes critical.

Here, we determined KDM4A-C subcellular localization during mitosis. We reveal a previously unrecognized differential mitotic localization of KDM4A-C members. While KDM4C is associated with mitotic chromatin, KDM4A-B proteins are excluded from chromatin throughout prometaphase–telophase. Also, we show that KDM4C Tudor domains are essential for its association with mitotic chromatin, thus characterizing a novel function of the Tudor domains in recruiting protein to mitotic chromatin. In addition, we demonstrate that dysregulation of KDM4C demethylase activity, but not KDM4B, promotes chromosome instability (CIN) by impairing the fidelity of mitotic chromosome segregation. These observations suggest that CIN, found in cancer cells driven by KDM4C dysregulation, could result from mitotic chromosome missegregation.

## MATERIALS AND METHODS

### Plasmid construction and domain swapping

The constructions of all plasmids used in this study are described in Table 1. All point mutations were introduced using the QuickChange site-directed mutagenesis kit (Stratagene). A complete list of all primers and their sequences is described in Table 2. Domain swapping was performed as described in Table 1. All constructs used in this study were verified by nucleotide sequencing or restriction digestion.

**Table 1.** Plasmids constructed in this study

Chimera	Plasmid name	Vector backbone	Insert
	pEGFP-N1-KDM4C1-708aa	pEGFP-N1 digested with *NheI*, *XhoI*	KDM4C1-708aa was amplified from pEGFP-N1-Hs-KDM4C-wt using F1 and R1
Chimera 1	pEGFP-N1-KDM4C-1-708-KDM4A-707-1064 aa	pEGFP-N1-KDM4C1-708aa digested with XhoI, SalI	KDM4A-707-1064 aa was amplified from pEGFP-N1-Hs-KDM4A-wt using F3 and R3
	pEGFP-N1-KDM4A1-714aa	pEGFP-N1 digested with *SalI*, *AfeI*	KDM4A1-714aa was amplified from pEGFP-N1-Hs-KDM4A-wt using F2 and R2
Chimera 2	pEGFP-N1-KDM4A-1-714-KDM4C-695-1056 aa	pEGFP-N1-KDM4A1-714aa digested with SalI, XmaI	KDM4C-695-1056 aa was amplified from pEGFP-N1-Hs-KDM4C-wt using F4 and R4
	pEGFP-N1-Hs-KDM4C1-865aa	pEGFP-N1 digested with *NheI*, *XhoI*	KDM4C1-865aa was amplified from pEGFP-N1-Hs-KDM4C-wt using F1 and R5
Chimera 3	pEGFP-N1-Hs-KDM4C1-865-KDM4A886-1064aa	pEGFP-N1-Hs-KDM4C1-865aa digested with XhoI, SalI	KDM4A886-1064aa was amplified from pEGFP-N1-Hs-KDM4A-wt using F6 and R3
	pEGFP-N1-KDM4A1-885aa	pEGFP-N1 digested with *SalI*, *AfeI*	KDM4A1-885aa was amplified from pEGFP-N1-Hs-KDM4A-wt using F2 and R6
Chimera 4	pEGFP-N1-KDM4A1-885-KDM4C866-1056aa	pEGFP-N1-KDM4A1-885aa digested with SalI, XmaI	KDM4C866-1056aa was amplified from pEGFP-N1-Hs-KDM4C-wt using F7 and R4
	pEGFP-N1-KDM4C1-934	pEGFP-N1 digested with *NheI*, Xho*I*	KDM4C1-934 was amplified from pEGFP-N1-Hs-KDM4C-wt using F1 and R7
Chimera 5	pEGFP-N1-KDM4C1-934-KDM4A935-1064aa	pEGFP-N1-KDM4C1-934 digested with *XhoI, SalI*	KDM4A935-1064aa was amplified from pEGFP-N1-Hs-KDM4A-wt using F8 and R3
	pEGFP-N1-KDM4A1-954	pEGFP-N1 digested with *SalI*, *AfeI*	KDM4A1-954 was amplified from pEGFP-N1-Hs-KDM4A-wt using F2 and R8
Chimera 6	pEGFP-N1-KDM4A1-954-KDM4C955-1056aa	pEGFP-N1-KDM4A1-954 digested with *SalI and XmaI*	KDM4C955-1056aa was amplified from pEGFP-N1-Hs-KDM4C-wt using F9 and R4
	pEGFP-N1-KDM4C-RTDF-DNLY	pEGFP-N1-KDM4C-WT	PCR mutagenesis using primers F10 and R10
	pEGFP-N1-KDM4A-DNLY-RTDF	pEGFP-N1-KDM4A-WT	PCR mutagenesis using primers F11 and R11
	pEGFP-N1-KDM4C-R919D	pEGFP-N1-KDM4C-WT	PCR mutagenesis using primers F12 and R12

**Table 2.** Primers used in this study

#	Name	Sequence
F1	SpeI-KDM4C-F1	GTACTAGTATGGAGGTGGCCGAGGTGGAA
R1	XhoI-KDM4C-R2121	ACTTGTTCCATCCTCCTCGAGGAAGGCATTGGGTGGAG
F2	Eco47III-D2A-F	GAGCCTCAGCGCTATGGCTTCTGAGTCTGAAACTCTGAAT
R2	SalI-KDM4A-R2150	CTGCAGCCAGTCGACGTGAAGCACATTTCTGGAATC
F3	XhoI-KDM4A-F2181	TCTACTCCTTATCTCGAGGAGGATGGCACCAGCATAC
R3	SalI-D2A-R	CATGTCGACCGCTCCATGATGGCCCGGTATAGTGCAG
F4	SalI-KDM4C-F2083	GCGGTCGACCGAAGAAAATATAGAATATTCTCCACCCAATG
R4	XmaI-D2C-R	ATCCCGGGTCTGTCTCTTCTGGCACTTCTTCTGGAAA
F5	SalI-KDM4C-F2083	GCGGTCGACCGAAGAAAATATAGAATATTCTCCACCCAATG
R5	XhoI-KDM4C-R2595	GGCTCGAGCTTATGTCGAAAGCATGTAATGTTCACCAC
F6	XhoI-KDM4A-F2656	GACTCGAGATTCCTAATTTGGAGCGTGCC
R6	SalI-KDM4A-R2655	AGGGTCGACCTTGTGCCGAAAGCAGGTAATGAAG
F7	SalI-KDM4C-F2598	ACGGTCGACGACAACCCCAACGTGAAGTCCAAGG
R7	XhoI-KDM4C-R2802	GGCTCGAGCAGCTTCAGACAGTCTCGGCTCACGATATC
F8	XhoI-KDM4A-F2865	GACTCGAGACTCCTCCTGCTGAAGGGGAAGT
R8	SalI-KDM4A-R2862	AGGGTCGACAAACTGGAGACAGTCCTGGCTCACTA
F9	SalI-KDM4C-F2805	ACGGTCGACGACCCACCTGCTGAGGGAGAAGTCGTC
F10	BamHI-KDM4C-RDTF-DNLY-F	GTTTGATGATGGATCCTTTAGCGATAACTTATATCCTGAGGATATCGTG
R10	BamHI-KDM4C-RDTF-DNLY-R	CACGATATCCTCAGGATATAAGTTATCGCTAAAGGATCCATCATCAAAC
F11	BamHI-KDM4A-DNLY-RTDF-F	ACTTTGATGATGGATCCTTCAGCCGCGATACCTTTCCTGAGGACATAGT
R11	BamHI-KDM4A-DNLY-RTDF-R	ACTATGTCCTCAGGAAAGGTATCGCGGCTGAAGGATCCATCATCAAAGT
F12	BamHI-KDM4C-R919D-F	TGTTTGATGATGGATCCTTTAGCGACGACACATTTCCTGAGGAT
R12	BamHI-KDM4C-R919D-R	ATCCTCAGGAAATGTGTCGTCGCTAAAGGATCCATCATCAAACA
R13	EcoR571-KDM4C-S198M	GCATGGCACACTGAAGACATGGACCTCTATATGATTAATTATCTCCAC

### Generation of stable cell lines

U2OS-TetON stable cell lines that conditionally express the fusions EGFP-KDM4B and EGFP-KDM4C were established as previously described (73). Cell line expressing EGFP-KDM4A was generated as follows. A fragment including EGFP-KDM4A was subcloned into pTRE2-puro (Clontech). The resulting pTRE2-puro-Hs-KDM4A vector was transfected into U2OS-Tet-ON cells (Clontech). Puromycin-resistant clones (0.6 μg/ml Puromycin) were selected and tested for doxycycline-induced expression of EGFP using fluorescence microscopy. Clones that showed EGFP expression only after the addition of 1 μM doxycycline (Sigma, D9891) were selected for further characterizations.

### Cell lines and growth conditions

All cells lines were supplemented with 10% heat-inactivated fetal bovine serum, 2 mM L-glutamine, 100 μg/ml penicillin, 100 μg/ml streptomycin and grown in a humidified incubator containing 5% CO_2_ at 37°C. U2OS cells were grown in DMEM. U2OS-Tet-ON cells were grown in Dulbecco's modified Eagle's medium (DMEM) in the presence of 200 μg/ml Geneticin (G418). U2OS-Tet-ON-EGFP-KDM4A-C cells were grown in DMEM containing 200 μg/ml G418 and either 0.6 μg/ml Puromycine (for KDM4A-B) or 200 μg/ml Hygromycin B (for KDM4C).

### Transient transfection

Cell transfections with plasmid DNA or siRNA were performed using Poly Jet (Bio-Consult) and Lipofectamine 2000 (Invitrogen), respectively, following the manufacturer's instructions. siRNAs used in this study include Stealth KDM4B-C siRNA (Invitrogen) and Stealth RNAi negative control. All constructs and siRNA sequences are available upon request.

### Western blotting

Protein lysates were prepared using two different methodologies. First, cells were lysed using NP40 lysis buffer (50 mM HEPES pH 7.4, 100 mM NaCl, 0.5% NP-40, 10 mM EDTA, 20 mM β-glycerophosphate, 0.1 mg/ml PMSF, 1.2 mM NaVO4, 5 mM NaF, 1 mM DTT, protease inhibitor cocktail and 25 μg/ml Benzonase (Novagen)) for 30 min on ice, centrifuged at 14 000  rpm for 25  min at 4°C, and supernatant was recovered. Second, protein lysates were prepared using hot-lysis buffer (1% SDS, 5mM EDTA, 50 mM Tris, pH7.5), boiled for 15 min, sonicated with two 15 s pulses of 35% amplitude, and centrifuged at 14 000 rpm for 15 min at room temperature; supernatant was then treated with benzonaze for 30 min, centrifuged and recovered. Protein concentration was determined using bicinchoninic acid (BCA) protein determination reagent (Sigma). Immunoblots were performed using appropriate antibodies (see Table 3). Membranes were developed using Quantum ECL detection kit (K-12042-D20, Advansta).

**Table 3.** Antibodies used in this study

Name	Source	Dilution for western blot	Dilution for IF
Primary antibodies			
Anti-H3K9me3	Abcam ab8898	1:2000	1:500
Anti-β-actin	SIGMA #A5441	1:15 000	
Anti-H3K36me3	Abcam ab9050	1:3000	
Anti-H3K4me3	Abcam ab8580	1:3000	
Anti-H3	Abcam ab1791	1:10 000	
Anti-KDM4C	Santa Cruz #sc-98678	1:1000	
Anti-KDM4C	Novus NBP149600		1:200
Anti-JMJD2B (KDM4B)	Santa Cruz sc-67192	1:1000	1:400
Anti-KDM4A	Abcam ab104831		1:250
Anti-GFP	Abcam ab290	1:1500	
Anti-α-tubulin	Santa Cruz #sc-23948		1:500
Anti-Pericentrin	Abcam ab4448		1:500
Secondary antibodies			
Donkey anti-mouse-Alexa Flour®488	Invitrogen #A21202		1:500
Donkey anti-rabbit-Alexa Flour®488	Invitrogen #A21206		1:500
Donkey anti-mouse-Alexa Flour®568	Invitrogen #A10037		1:500
Donkey anti-rabbit-Alexa Flour®568	Invitrogen #A10042		1:500
Donkey anti-rabbit DyLightTM649	Jackson ImmunoResearch		1:500
Anti-mouse(IgG)-HRP	Amersham	1:10 000	
Anti-rabbit(IgG)-HRP	Jackson ImmunoResearch #111-035-003	1:20 000	

### Immunofluorescence and Microscopy

Cells were grown on coverslips for 24–48 h before fixation and then washed twice with PBSX1, fixed with 4% paraformaldehyde for 10 min, permeabilized with 0.15% Triton-X-100 and 0.15% Tween-20 in PBSx1 for 10 min, blocked with 3% BSA, 0.2% Tween-20 and 0.2% Triton-X-100 for 1 h at RT, stained with the appropriate primary antibodies (see Table 3 for a complete list of all antibodies used in this study) for 3 h at 37°C, washed three times with wash buffer (0.2% Tween-20 and 0.2% Triton-X-100 in PBSx1), stained with AlexaFluor488, AlexaFluor568 (Molecular Probes; 1:500) or DyLight 649 (Jackson ImmunoResearch; 1:500) secondary antibodies for 1 h at RT in dark and then washed as above. Slides were then mounted using VECTASHIELD mounting medium with DAPI (VECTOR) and photographed using an inverted microscope Confocal Zeiss LSM 700 with 40X oil EC Plan Neofluar objective.

## RESULTS

### KDM4C, but not KDM4A-B, protein is associated with mitotic chromatin

**Figure 1.**
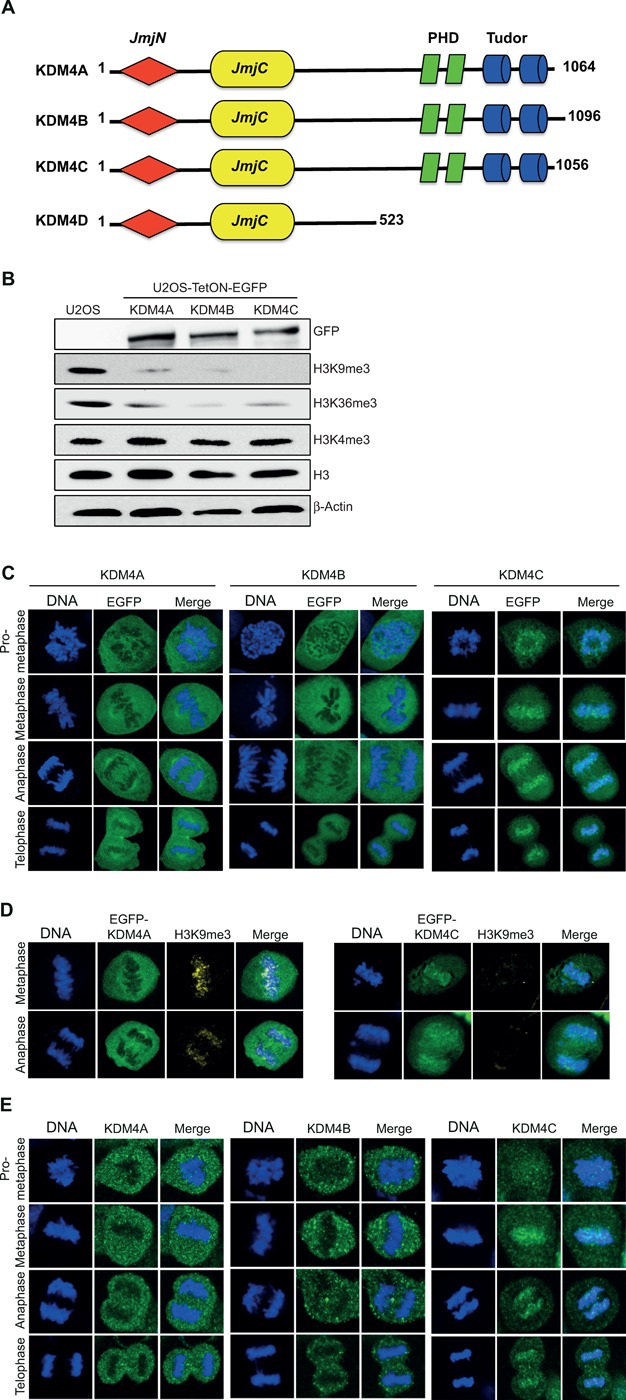
Differential localization of EGFP-KDM4A-C fusions during mitosis. (**A**) Schematic structure representation of the KDM4A-D histone demethylases. The KDM4 family consists of four members: KDM4A, KDM4B, KDM4C and KDM4D. All members, except KDM4D, share, in addition to JmjC and JmjN, two PHD and two Tudor domains. KDM4A-C schematic was built using the SMART software (http://smart.embl-heidelberg.de). (**B**) Western blot analysis shows that U2OS-TetON cells express comparable levels of KDM4A-C proteins that demethylate H3K9me3 and H3K36me3 but not H3K4me3. Cells were treated with Dox for 36 h and protein lysates were prepared using hot-lysis and immunoblotted with the indicated antibodies. (**C**) Representative images showing the localization of EGFP-KDM4A-C fusions from prometaphase to telophase. U2OS-Tet-ON-EGFP-KDM4A-C cells were treated with Dox to induce the expression of EGFP-KDM4A-C fusions (green). Cells were stained with DAPI (blue). KDM4A and KDM4B are excluded from chromatin while KDM4C is associated with mitotic chromatin. Twenty to thirty cells were counted for each of the mitotic stages expressing either KDM4A or KDM4B. 0ver 100 cells were counted for mitotic cells expressing EGFP-KDM4C. (**D**) Overexpression of EGFP-KDM4C, but not EGFP-KDM4A, leads to a severe reduction in H3K9me3 levels. U2OS-Tet-ON-EGFP-KDM4A and U2OS-Tet-ON-EGFP-KDM4C cells were treated with Dox for 36 h, fixed and subjected to immunofluorescence analysis using H3K9me3 antibody (yellow). DNA is stained with DAPI (blue) and EGFP-KDM4A and EGFP-KDM4C are in green. Results shown in (C) and (D) are typical of at least two independent experiments. (**E**) Representative images showing the localization of the endogenous KDM4A-C proteins from prometaphase to telophase. MCF7 cells were fixed and subjected to immunofluorescence analysis using KDM4A-C antibodies.

Revealing the subcellular localization of proteins is vital for understanding their biological function(s). We sought therefore to determine the localization of KDM4A-C members, which share common domain architecture consisting of JmjN, JmjC, two PHD and two Tudor domains (Figure 1A). Toward this, we established U2OS-TetON cell lines expressing comparable protein levels of EGFP-KDM4A-C fusions upon the addition of doxycycline (Dox). As shown in Figure 1B, addition of Dox induces the expression of EGFP-KDM4A-C fusions, which leads to a severe reduction in H3K9me3 and H3K36me3 levels. On the other hand, the H3K4me3 levels were not affected by overexpression of KDM4A-C proteins.

To determine the localization of EGFP-KDM4A-C fusions during the different stages of mitosis, U2OS-TetON-EGFP-KDM4A-C cells were treated with Dox for 36 h, fixed and stained with DAPI to visualize mitotic cells. Results show that KDM4C protein is localized to mitotic chromatin from prometaphase to telophase. In striking contrast to KDM4C, EGFP-KDM4A-B fusions are excluded from mitotic chromatin (Figure 1C). Interestingly, the levels of H3K9me3 on mitotic chromatin are severely reduced in cells overexpressing EGFP-KDM4C comparing to cell overexpressing EGFP-KDM4A fusion (Figure 1D).

Next, to assess whether the mitotic localization of the endogenous KDM4A-C proteins is similar to their overexpressed EGFP-fused forms, we first tested the suitability of commercial KDM4A-C antibodies to detect the native forms of KDM4A-C proteins by immunofluorescence analysis. U2OS cells were transfected with expression constructs expressing EGFP-KDM4A-C fusions (green) and immunostained with KDM4A-C antibodies (red). Results show that the intensity of the red signal in cells expressing the EGFP-KDM4A-C fusions is much higher than the untransfected cells (Supplementary Figure S1). This result confirms that these antibodies detect KDM4A-C proteins in cells and can be used for immunofluorescence-based studies. MCF7 cells were then immunostained using KDM4A-C antibodies to detect their mitotic localization. Results show that, similar to the localization of EGFP-KDM4A-C fusions, the endogenous KDM4A-B are excluded from mitotic chromatin, while KDM4C protein is associated with chromatin during the different mitotic stages (Figure 1E). Altogether, these observations demonstrate for the first time that, unlike KDM4A and KDM4B, KDM4C is associated with mitotic chromatin and triggers the demethylation of H3K9me3 mark.

### The Tudor domains of KDM4C mediate its localization to mitotic chromatin

**Figure 2.**
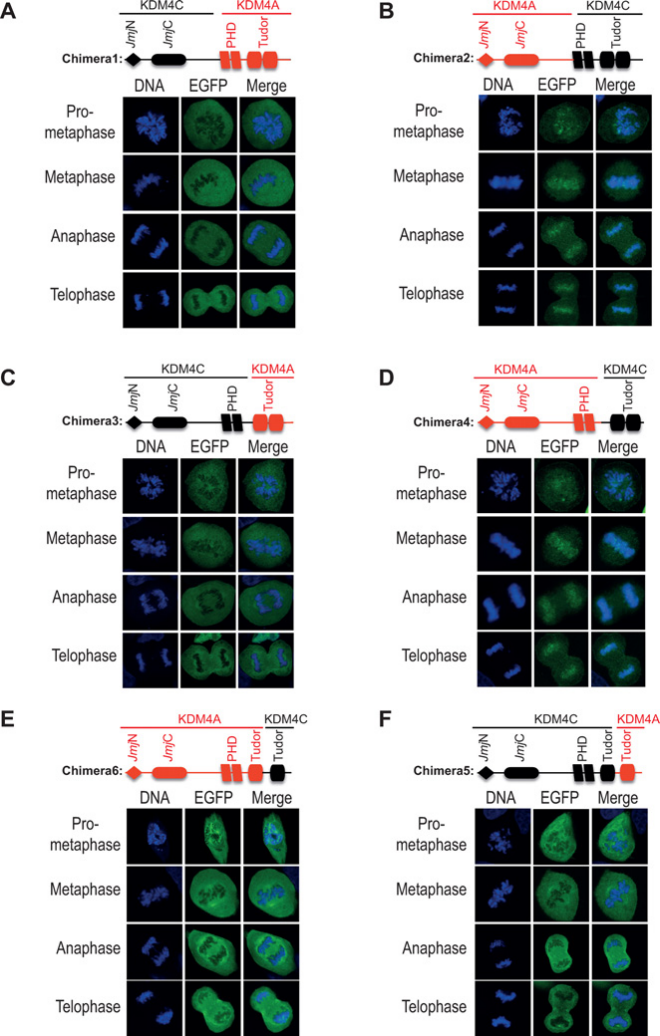
The C-terminal region of KDM4C mediates its association with mitotic chromatin. Panels (**A**)**–**(**D**) show that the C-terminus of KDM4C, containing the two Tudor domains, is essential and sufficient for its association with mitotic chromatin. Panels (**E**)**–**(**F**) show that the distal Tudor domain is essential but not sufficient for the localization of KDM4C at mitotic chromatin. In panels (A)–(F), U2OS cells were transfected with expression constructs encoding the indicated chimeras fused to EGFP (green). DNA is stained with DAPI (blue). Results are typical of 2–3 different experiments and each image represents at least 10 different cells. Image acquisition and scoring were performed by a student who was blind of the experimental condition.

To map KDM4C region that mediates its localization to mitotic chromatin, we performed domain-swapping analysis between KDM4A and KDM4C proteins. First, we swapped the regions containing the two PHD and the two Tudor domains between KDM4A and KDM4C proteins. As a result, two chimeras were produced (Table 1): chimera1 encodes the first 708 amino acids of KDM4C protein, which includes the JmjN and JmjC domain, fused to the last 357 amino acids of the KDM4A containing the two PHD and the two Tudor domains. Chimera2 is the reciprocal chimera, which encodes the first 714 amino acids of KDM4A protein and the last 361 amino acids of the KDM4C protein containing the two PHD and the two Tudor domains. U2OS cells were transfected with expression vectors encoding chimera1 and 2 and the mitotic localization was determined as described in Figure 1B. Results show that while chimera1 shows chromatin-excluded localization (Figure 2A), chimera2 exhibits chromatin-bound localization during mitosis (Figure 2B). Together, these results suggest that the C-terminal region containing the two PHD and the two Tudor domains mediate the distinct mitotic localization of KDM4A and KDM4C proteins.

To identify the domain that mediates the association of KDM4C with mitotic chromatin, we repeated the domain-swapping analysis and exchanged the regions containing only the two Tudor domains between KDM4A and KDM4C proteins. This analysis produced chimera3 and chimera4 (Table 1). Chimera3 encodes the first 865 amino acids of KDM4C protein and the last 178 amino acids of KDM4A that contain the two Tudor domains. Chimera4 encodes the first 885 amino acids of KDM4A protein and the last 190 of KDM4C protein. We observed that chimera3 shows chromatin-excluded localization (Figure 2C) and chimera4 has chromatin-bound localization during mitosis (Figure 2D). These results show that the replacement of KDM4C Tudor domains with those of KDM4A protein leads to its exclusion from mitotic chromatin. In addition, KDM4C C-terminal region, consisting of the two Tudor domains, leads to the association of KDM4A with mitotic chromatin. Collectively, we concluded that the KDM4C C-terminus, containing the two Tudor domains, is essential and sufficient for its association with mitotic chromatin.

To determine whether both Tudor domains are required for KDM4C mitotic localization, we swapped the C-terminus region containing the distal Tudor domain between KDM4C and KDM4A (Table 1). Chimera5, which encodes the first 934 amino acids of KDM4C fused with the last 129 amino acid containing the distal Tudor domain of KDM4A, is excluded from mitotic chromatin (Figure 2E). On the other hand, chimera6 that encodes the first 954 amino acids of KDM4A fused to 101 amino acids of KDM4C, which includes its distal Tudor domain, remains excluded from chromatin (Figure 2F). We concluded therefore that the C-terminus of KDM4C containing the distal Tudor domain is essential but not sufficient for its mitotic chromatin localization.

### Mapping candidate residues within KDM4C Tudor domains that regulate its localization to mitotic chromatin

**Figure 3.**
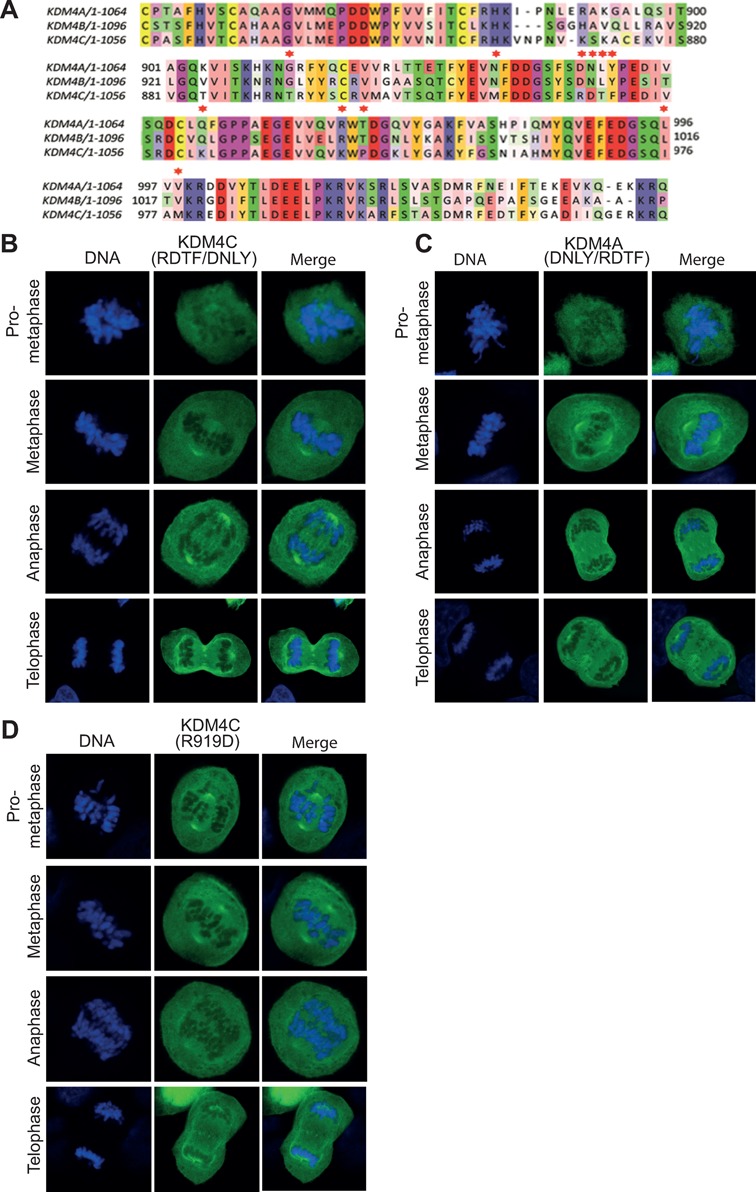
KDM4C-R919 residue is essential but not sufficient for KDM4C association with mitotic chromatin. (**A**) Multiple sequence alignment of the KDM4A-C Tudor domains was performed using the MUSCLE software. The color-coding is based on the Zapo color scheme. Black arrowhead indicates amino acid residues that are conserved in KDM4A and KDM4B isoforms, and are distinct in KDM4C. (**B**)**–**(**D**) U2OS cells were transfected with expression constructs encoding the indicated KDM4A and KDM4C mutants fused to EGFP. Results are typical of two independent experiments and each image represents at least 10 different cells.

Domain-swapping analyses suggest that the localization of KDM4C at mitotic chromatin is mediated by its Tudor domains (Figure 2). To map residues within the Tudor domains of KDM4C that regulate its association with mitotic chromatin, we performed sequence alignment of KDM4A-C proteins using MUSCLE software and searched for residues that are conserved between KDM4A and KDM4B proteins but not in KDM4C (Figure 3A). Noticeably, comparison of the amino acid sequences shows that the proximal Tudor domain of KDM4A and KDM4B contains four identical amino acids, DNLY, which appear as RDTF in KDM4C protein (corresponds to 919–922 amino acids). On this basis, we speculated that these four residues might be implicated in regulating KDM4C localization at mitotic chromatin. Site-directed mutagenesis was used to substitute RDTF residues of KDM4C with DNLY, and the mitotic localization of KDM4C^RDTF/DNLY^ mutant was determined. Results show that EGFP-KDM4C^RDTF/DNLY^ mutant is excluded from mitotic chromatin (Figure 3B). We concluded therefore that the RDTF residues are critical for KDM4C association with mitotic chromatin. Next, we sought to address whether RDTF residues are sufficient for KDM4C localization at mitotic chromatin. To do so, we substituted the DNLY (corresponds to 939–942 amino acids) of KDM4A with RDTF. Results show that, similar to the wild-type KDM4A, KDM4A^DNLY/RDTF^ mutant remains excluded from mitotic chromatin (Figure 3C). Altogether, these observations suggest that RDTF residues are required, but not sufficient, for the localization of KDM4C at mitotic chromatin.

Interestingly, it was recently shown that KDM4A^D939^ residue is essential for the binding of the KDM4A Tudor domain with methylated H4K20me2 (56). Therefore, we sought to address whether this residue is also involved in regulating KDM4A and KDM4C association with mitotic chromatin. Toward this, we generated KDM4C^R919D^. Results show that this mutant is excluded from mitotic chromatin (Figure 3D). This observation suggests that, similar to the RDTF residues, KDM4C^R919^ residue is essential for KDM4C localization at mitotic chromatin. Collectively, our data strongly suggest that sequences at the two Tudor domains are required for the distinct localization of KDM4C at mitotic chromatin.

### Dysregulation of KDM4C expression promotes mitotic chromosome missegregation

**Figure 4.**
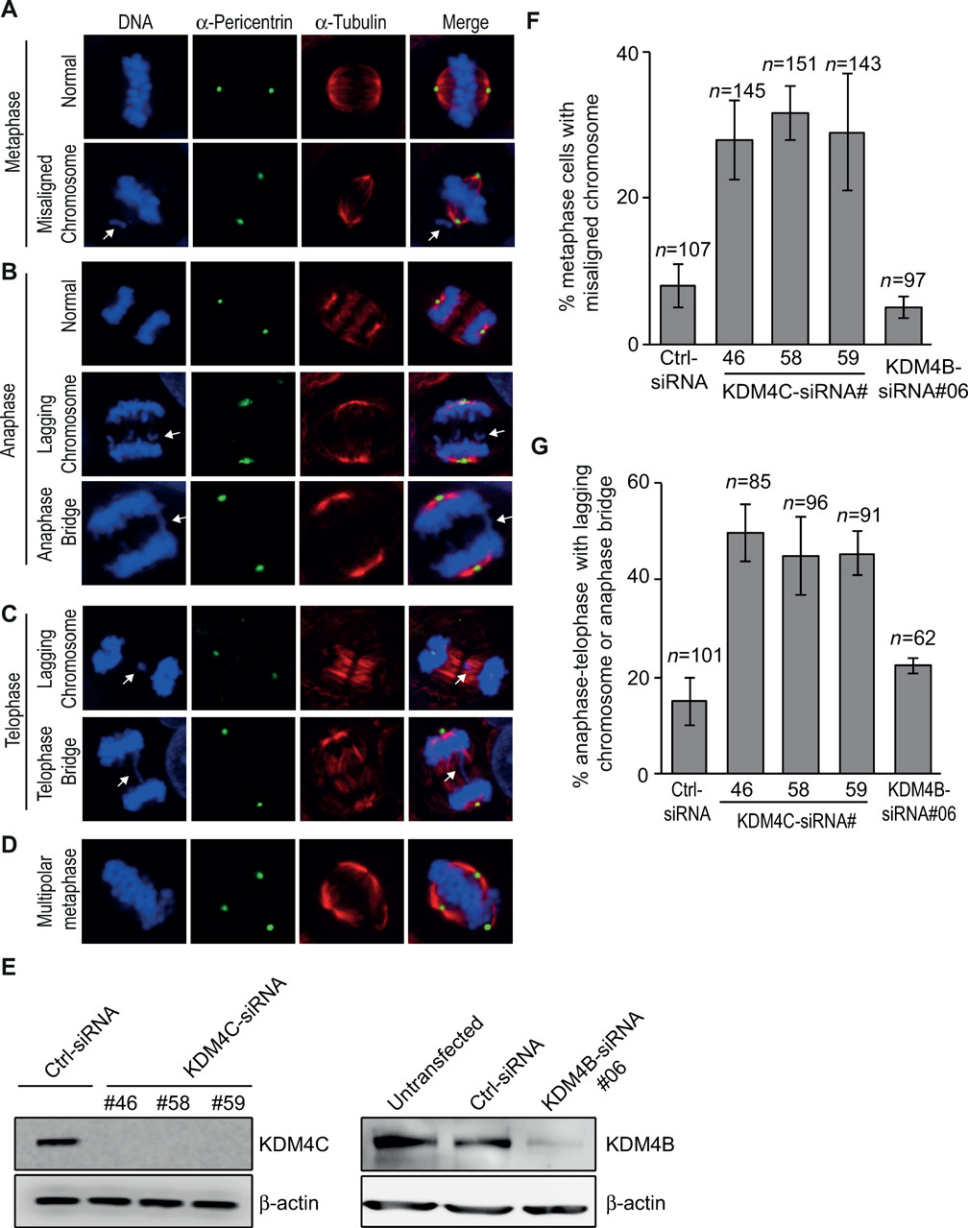
KDM4C, but not KDM4B, depletion increases chromosomal segregation errors during mitosis. (A)**–**(D) Representative images showing normal and defective mitotic U2OS cells depleted of KDM4C. Cells were subjected to immunofluorescence analysis using Pericentrin (green) and α-tubulin antibodies (red). DNA is stained with DAPI (blue). (**A**) Normal and abnormal metaphase with misaligned chromosome (indicated by white arrow). (**B**) Normal and abnormal anaphase with either lagging chromosomes or anaphase bridge (indicated by white arrows). (**C**) Abnormal telophase with either lagging chromosomes or telophase bridge (indicated by white arrows). (**D**) Multipolar metaphase. (**E**) KDM4B and KDM4C knockdown by western blotting. U2OS cells were transfected with either control or different sequences of KDM4B and KDM4C Stealth siRNA (Invitrogen). Protein extracts were prepared 72 h after transfection and immunoblotted with KDM4B and KDM4C antibody. β-Actin is used as a loading control. (**F**) A histogram showing the percentage of metaphases with misaligned chromosomes 72 h after transfection with control and different KDM4B-C siRNA sequences. KDM4C, but not KDM4B, depletion increases the frequency of metaphase cells with misaligned chromosomes. *n*, number of metaphase cells counted. Error bars represent standard deviation from two independent experiments. (**G**) KDM4C, but not KDM4B, depletion increases the frequency of anaphase–telophase cells with either lagging chromosomes or anaphase–telophase bridges. As in (F), except that the histogram shows the percentage of defective anaphase–telophase cells.

The localization of KDM4C on mitotic chromatin raises a possibility that it might be implicated in regulating chromosome segregation. To assess this possibility, we looked at four abnormal mitotic phenotypes in cells overexpressing or depleted of either KDM4B or KDM4C. The abnormal phenotypes include misaligned chromosomes during metaphase (Figure 4A), lagging chromosomes, anaphase–telophase bridges (Figure 4B and C) and multiple centrosomes (Figure 4D). To deplete KDM4B-C, U2OS cells were transfected with KDM4B-C siRNA sequences (KDM4C siRNA #46, #58 and #59; KDM4B #06). Western blot reveals that all siRNA sequences targeting KDM4B-C show severe reduction in the protein levels compared to control siRNA (Figure 4E).

KDM4C-depleted cells were subjected to immunofluorescence analysis using α-tubulin and α-Pericentrin antibodies and DNA was stained with DAPI to allow the identification of mitotic cells. Metaphase cells with misaligned chromosomes were counted for each KDM4C siRNA sequences and divided by the total number of metaphase cells (*n* = 448 total metaphases). Results show that KDM4C depletion using either one of the three siRNA sequences leads to over 3-fold increase in percentage of metaphase cells with misaligned chromosomes compared to cell transfected with control siRNA (*n* = 107 metaphase cells) (Figure 4F). Similar increase was also obtained in anaphase–telophase cells with lagging chromosomes or anaphase–telophase bridges (*n* = 272 anaphase–telophase cells) compared to control cells (*n* = 101) (Figure 4G). Next, we sought to address whether KDM4C depletion affects centrosome number. Mitotic cells were analyzed based on α-tubulin and Pericentrin staining. Results show no detectable changes in the percentage of mitotic cells with multiple spindle poles between control and KDM4C-depleted cells (multipolar spindle pole formation was present in 0.5±0.4% of KDM4C-depleted cells and 0.4±0.3% of control cells). Interestingly, KDM4B-depleted cells show no significant increase in the percentage of abnormal mitotic cells (Figure 4F and G). Altogether, these observations show for the first time that depletion of KDM4C, but not KDM4B, affects the fidelity of mitotic chromosome segregation, therefore suggesting that CIN in cancers lacking KDM4C can result in part from mitotic chromosome missegregation.

**Figure 5.**
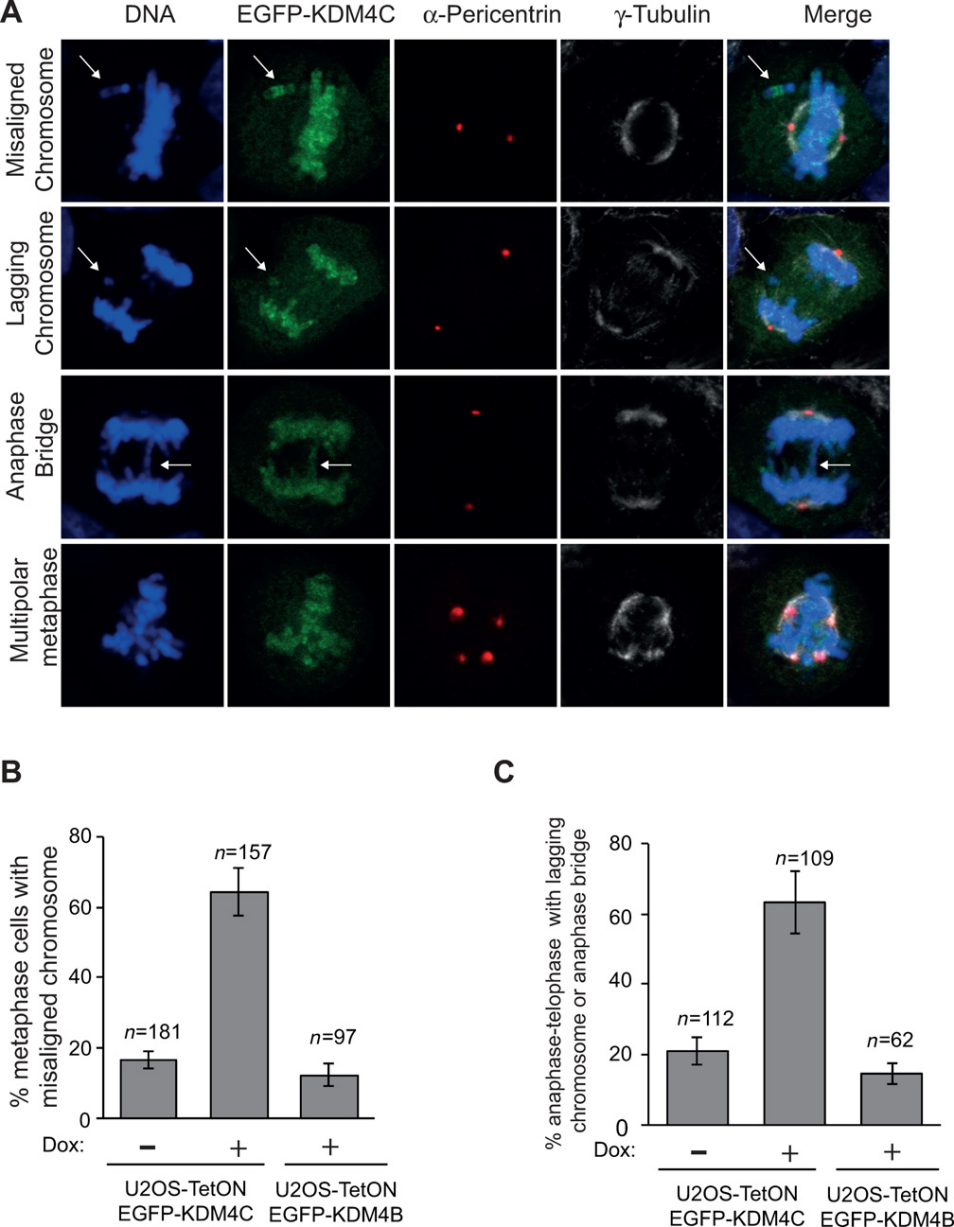
KDM4C, but not KDM4B, overexpression promotes mitotic chromosome missegregation. (**A**) Representative images of defective mitotic cells overexpressing EGFP-KDM4C fusion. U2OS-TetON-EGFP-KDM4C cells were treated with Dox for 72 h to induce the expression of EGFP-KDM4C (green). Cells were then stained for Pericentrin (red) and α-tubulin antibodies (gray). DNA is stained with DAPI (blue). (**B**) and (**C**) Histograms showing the percentage of metaphases with misaligned chromosomes (B) and anaphase–telophase cells that exhibit lagging chromosomes or anaphase–telophase bridges (C). Untreated and Dox-treated U2OS-TetON-EGFP-KDM4C and U2OS-TetON-EGFP-KDM4B cells were subjected to immunofluorescence and mitotic cells were acquired using confocal microscope. *n*, number of mitotic cells counted. Error bars represent standard deviation from three and two independent experiments of cells expressing KDM4C and KDM4B, respectively.

Importantly, KDM4B-C members are overexpressed in several types of human cancer and its depletion impairs cancer cell proliferation (24,50,61,64–69,71,74,75). We sought therefore to address whether KDM4B-C overexpression also affects mitotic chromosome missegregation. To do so, U2OS-TetON cells were treated with Dox for 72 h to induce the expression of EGFP-KDM4C or EGFP-KDM4B fusions. Cells were then fixed and stained with α-tubulin, α-Pericentrin and DAPI to determine the frequency of abnormal mitotic cells. Results show that Dox treatment of U2OS-TetON-EGFP-KDM4C cells leads to 3.8-fold increase in metaphase cells with misaligned chromosomes (Figure 5A) compared to Dox-untreated cells (Figure 5B). Likewise, the frequency of cells with lagging chromosomes and anaphase–telophase bridges (Figure 5A) was increased by 3-fold following the expression of EGFP-KDM4C fusion (Figure 5C). We concluded that similar to KDM4C knockdown, upregulation of KDM4C promotes mitotic chromosome missegregation that can potentially lead to CIN found in cancer driven by KDM4C overexpression. Similar to KDM4C depletion, no significant changes were observed in the frequency of multipolar mitotic cells after the addition of Dox (Dox-untreated cells show 0.5±0.3% and Dox-treated cells show 0.5±0.4%). Importantly, the increase in the percentage of abnormal mitotic cells was not observed in cells overexpressing EGFP-KDM4B fusion (Figure 5B and C). Collectively, our data suggest that both up- and downregulation of KDM4C have similar effect on the fidelity of mitotic chromosome segregation.

### The demethylase activity and the mitotic localization of KDM4C influence the integrity of mitotic chromosome segregation

**Figure 6.**
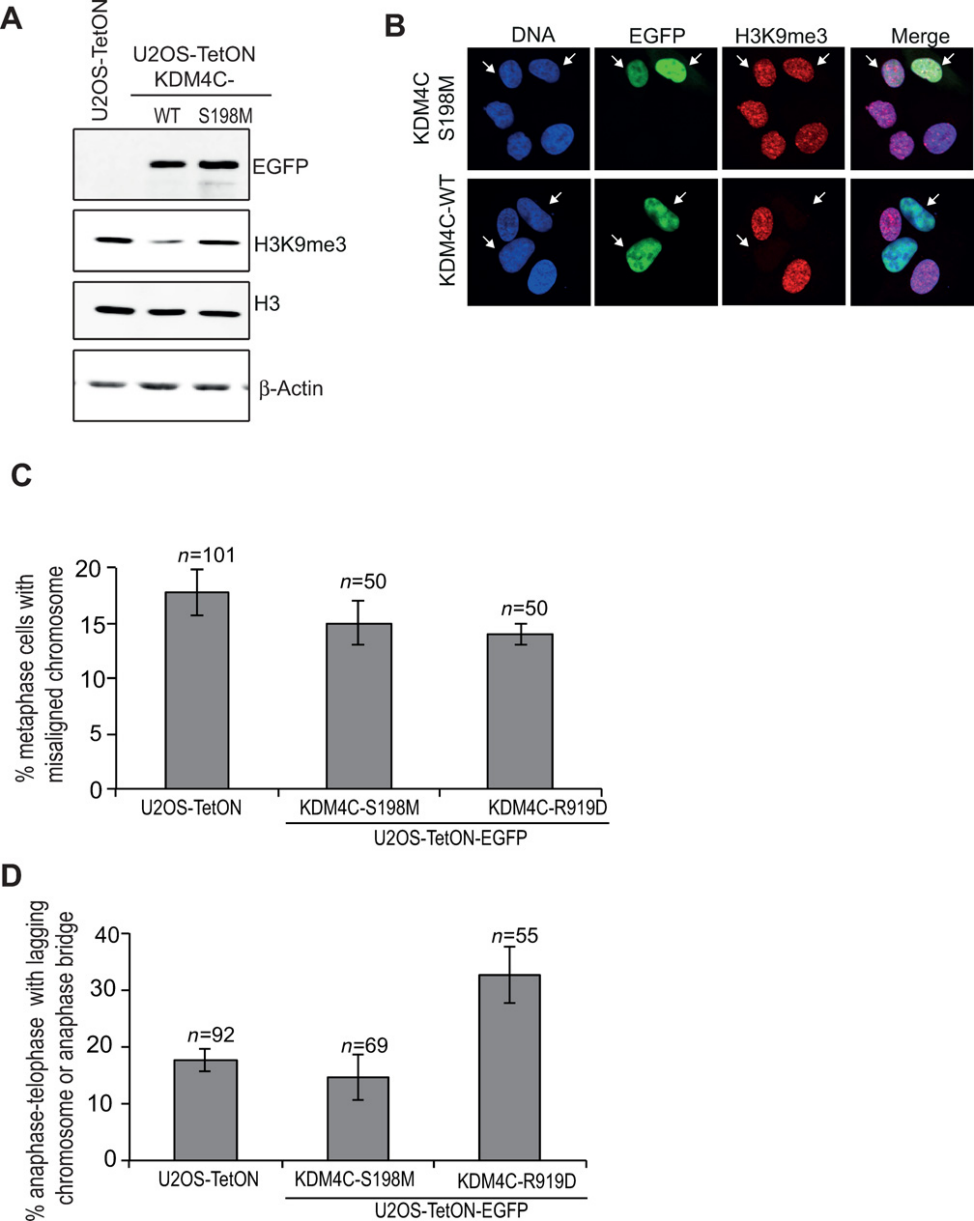
KDM4C demethylase activity and mitotic localization affect the fidelity of chromosome segregation during mitosis. (**A**) and (**B**) show the effect of S198M mutation on the demethylase activity of KDM4C protein. (A) western blot analysis showing that overexpression of EGFP-KDM4C-S198M has no detectable effect on the levels of H3K9me3. Protein extracts were prepared from U2OS-TetON cells expressing either EGFP-KDM4C-WT or EGFP-KDM4C-S198M and immunoblotted using the indicated antibodies. (B) Immunofluorescence analysis of U2OS-TetON expressing either EGFP-KDM4C-WT (bottom) or EGFP-KDM4C-S198M (top). Cells were stained for H3K9me3 (red). DNA is stained with DAPI (blue), and the EGFP-KDM4C is in green. (**C**) and (**D**) Histograms showing the percentage of metaphases with misaligned chromosomes (B) and anaphase–telophase cells that exhibit lagging chromosomes or anaphase–telophase bridges (C). U2OS-TetON cells expressing EGFP-KDM4C-S198M or EGFP-KDM4C-R919D were subjected to immunofluorescence and mitotic cells were acquired using confocal microscope. *n*, number of mitotic cells counted. Error bars represent standard deviation from two independent experiments.

To gain further insights into KDM4C mitotic function, we sought to address whether its demethylase activity is implicated in regulating chromosome segregation. Toward this, we generated KDM4C ‘demethylase-dead’ mutant. As we have previously reported (76), Ser198Met mutation, within the JmjC of KDM4C, is expected to abolish an existing hydrogen-bond network, disrupting the coordination of α-KG within the catalytic site and consequently abrogating the demethylase activity. Indeed, western blot and immunofluorescence analysis show that overexpression of KDM4C-S198M in U2OS cells has no effect on H3K9me3 levels, whereas overexpression of wild-type KDM4C leads to a sever decrease in the levels of H3K9me3 mark (Figure 6A and B). Next, we looked at abnormal mitosis in cells overexpressing EGFP-KDM4C-S198M demethylase-dead mutant. Results show no detectable effect on the percentage of cells showing abnormal chromosome segregation (Figure 6C and D). This observation suggests that dysregulation of KDM4C demethylase activity disrupt the fidelity of mitotic chromosome segregation.

Next, we sought to address whether the defective chromosome segregation in cells overexpressing KDM4C is due to its localization on mitotic chromatin. To this end, we overexpressed EGFP-KDM4C-R919D mutant, which is not associated with mitotic chromatin (Figure 3D), and determined the percentage of abnormal mitotic cells. Results show that EGFP-KDM4C-R919D overexpression has no significant increase in the percentage of metaphases with misaligned chromosomes (Figure 6C). On the other hand, it led to 1.8-fold increase in the percentage of anaphase–telophase cells showing lagging chromosomes or anaphase–telophase bridge (Figure 6D). Altogether, we made two interesting conclusions. The first is that the defective mitotic chromosome segregation in cells overexpressing or depleted of KDM4C is due to alteration in KDM4C demethylase activity. The second is that part of the abnormal mitotic phenotype (lagging chromosomes and anaphase–telophase bridges) is independent of its localization on mitotic chromatin.

## DISCUSSION

This study shows a unique localization of KDM4C on mitotic chromatin that differs from the closely related members, KDM4A and KDM4B, which are excluded from chromatin during mitosis (Figure 1). This differential mitotic localization of KDM4A-C suggests that they might have distinct functions during mitosis. Similar to our observations, it was reported that the three different isoforms of the heterochromatin protein (HP1α, HP1β and HP1γ), which share the same domain architecture, are localized differently during interphase and mitosis. HP1α is the only isoform that remains associated with chromatin during mitosis, while HP1β and HP1γ showed different mitotic distribution compared to their nuclear localization during interphase (77,78). In agreement with this, HP1α isoform plays a central role in mitotic chromosome segregation by regulating Aurora B activation and dissociation from chromosome arms (79). Furthermore, protein phosphatase-1 (PP-1) isoforms α, γ1 and δ, which share nearly identical catalytic domains, localize differently during interphase and mitosis suggesting unique roles for each of the PP-1 isoforms during the different cell-cycle stages (80).

Our data suggest that the two Tudor domains are involved in the regulation of KDM4C localization during mitosis. Interestingly, the Tudor domain was shown to mediate the subcellular localization of proteins. For example, the Tudor domain of TDRD3 is both required and sufficient for its localization to stress granules, which are cytoplasmic structures involved in RNA metabolism (81). Similarly, Tudor domain-containing protein, Yb, which is required for the primary processing of piRNAs and transposon repression, localizes to a cytoplasmic structure called the Yb body via its Tudor domain (49). Also, the Tudor domain mediates the recruitment of various proteins to chromatin. For instance, it was found that foci formation of 53BP1 protein after DNA damage is mediated by the binding of its Tudor domain to H4K20me2 mark (56,82). Moreover, it was shown that KDM4A is guided by its Tudor domain to H3K4me3 and H4K20me3 regions to demethylate H3K9me3 and H3K36me3 methyl marks (83,84). Here, we further expand the function of the Tudor domain by characterizing a previously unrecognized role of the Tudor domain in recruiting KDM4C to mitotic chromatin. Future work will be required to identify the mechanism by which the Tudor domains regulate KDM4C association with chromatin during mitosis.

In addition, we reveal a novel role of KDM4C in regulating mitotic chromosome segregation. Our results show that the levels of KDM4C protein are critical for the proper chromosome segregation. KDM4C, but not KDM4B, knockdown or overexpression increases the frequency of abnormal mitotic cells showing misaligned chromosomes during metaphase, anaphase bridge and chromosome lagging. Further, overexpression of KDM4C-S198M demethylase-dead mutant has no detectable effect on the fidelity of chromosome segregation. These results imply that CIN can result from loss or gain of KDM4C demethylase activity. In accord with this, recent analysis of the Cancer Genome Atlas revealed that KDM4C is lost in some cancer types and overexpressed in others (62). It should be noted however that we cannot exclude the existence of additional yet unknown mechanisms that contribute to CIN found in cancer driven by either lack or overactivity of KDM4C lysine demethylase. In this regard, it was recently shown that KDM4A overexpression induces copy number gains of specific genomic regions which are known to contain oncogenes (62).

Determining the cellular localization of protein is often a crucial step toward understanding its biological functions (85). Our data showing that KDM4C is localized at mitotic chromatin and promotes chromosome segregation is in accordance with the mitotic localization pattern of other proteins, which are associated with mitotic chromatin and play a role in chromosome condensation and sister chromatid separation. For example, the Tudor-domain protein EKL-1 is localized at mitotic chromatin and promotes chromosome segregation (86). Likewise, Aurora B kinase, which is associated with mitotic chromosomes, is involved in chromosome segregation and cytokinesis (87,88).

How does KDM4C misregulation disrupt the fidelity of mitotic chromosome segregation? There are three main possibilities which are not necessarily mutually exclusive. First, KDM4C serves as a scaffold protein for recruiting other proteins that are required for proper chromosome segregation. In accord with this, cells overexpressing KDM4C-R919D mutant (which does not localize to mitotic chromatin) show no increase in the percentage of metaphases with misaligned chromosome (Figure 6C). Second, KDM4C regulates the activity of non-histone proteins, which are involved in regulation of chromosome segregation, through demethylating their lysine residues. In support of this, it was shown that the polycomb protein, Pc2, is a substrate of KDM4C. Interestingly, Pc2 is SUMO E3 ligase that promotes sumoylation of multiple proteins (89), a modification which is essential for proper chromosomes segregation (90). Third, by demethylating KDM4C histone substrates such as methylated H3K9 or H3K36 residues. In agreement with this, it was shown that the level of H3K9me3 mark decreases as cells exit mitosis, during the period between anaphase and cytokinesis. This decrease is essential for chromosome congression and segregation. It was also found that H3K9me3-deficient cells exhibit a wide range of abnormal mitotic phenotypes such as an increase in misaligned and lagging chromosomes, which leads to aneuploidy, nondisjunction and the appearance of micronuclei at cytokinesis or early G1 (91). Likewise, it was previously shown that loss of H3K9 methyltransferase, Suv39h, or overexpression of H3K9 demethylase KDM4B leads to CIN (18,92). Collectively, our data suggest that definite methylation levels of KDM4C substrates might be required to ensure proper chromosome segregation. Nonetheless, further studies will be required to address how alterations in KDM4C levels promote chromosome missegregation.

## SUPPLEMENTARY DATA

Supplementary Data are available at NAR Online.

SUPPLEMENTARY DATA
