# Supplementary material for: KDM4C (GASC1) lysine demethylase is associated with mitotic chromatin and regulates chromosome segregation during mitosis
Source: Nucleic Acids Res. 2014 Apr 11;42(10):6168–82. doi: 10.1093/nar/gku253 (PMC4041427; doi:10.1093/nar/gku253)
Supplement: SUPPLEMENTARY DATA [file supp_42_10_6168__index.html]

KDM4C (GASC1) lysine demethylase is associated with mitotic chromatin and regulates chromosome segregation during mitosis — KDM4C (GASC1) lysine demethylase is associated with mitotic chromatin and regulates chromosome segregation during mitosis — SUPPLEMENTARY DATA 

# KDM4C (GASC1) lysine demethylase is associated with mitotic chromatin and regulates chromosome segregation during mitosis

## SUPPLEMENTARY DATA

**Files in this Data Supplement:**

- SUPPLEMENTARY DATA
