# Supplementary material for: KDM4C (GASC1) lysine demethylase is associated with mitotic chromatin and regulates chromosome segregation during mitosis
Source: Nucleic Acids Res. 2014 Apr 11;42(10):6168–82. doi: 10.1093/nar/gku253 (PMC4041427; doi:10.1093/nar/gku253)
Supplement: SUPPLEMENTARY DATA [file supp_gku253_nar-00239-x-2014-File009.pdf]

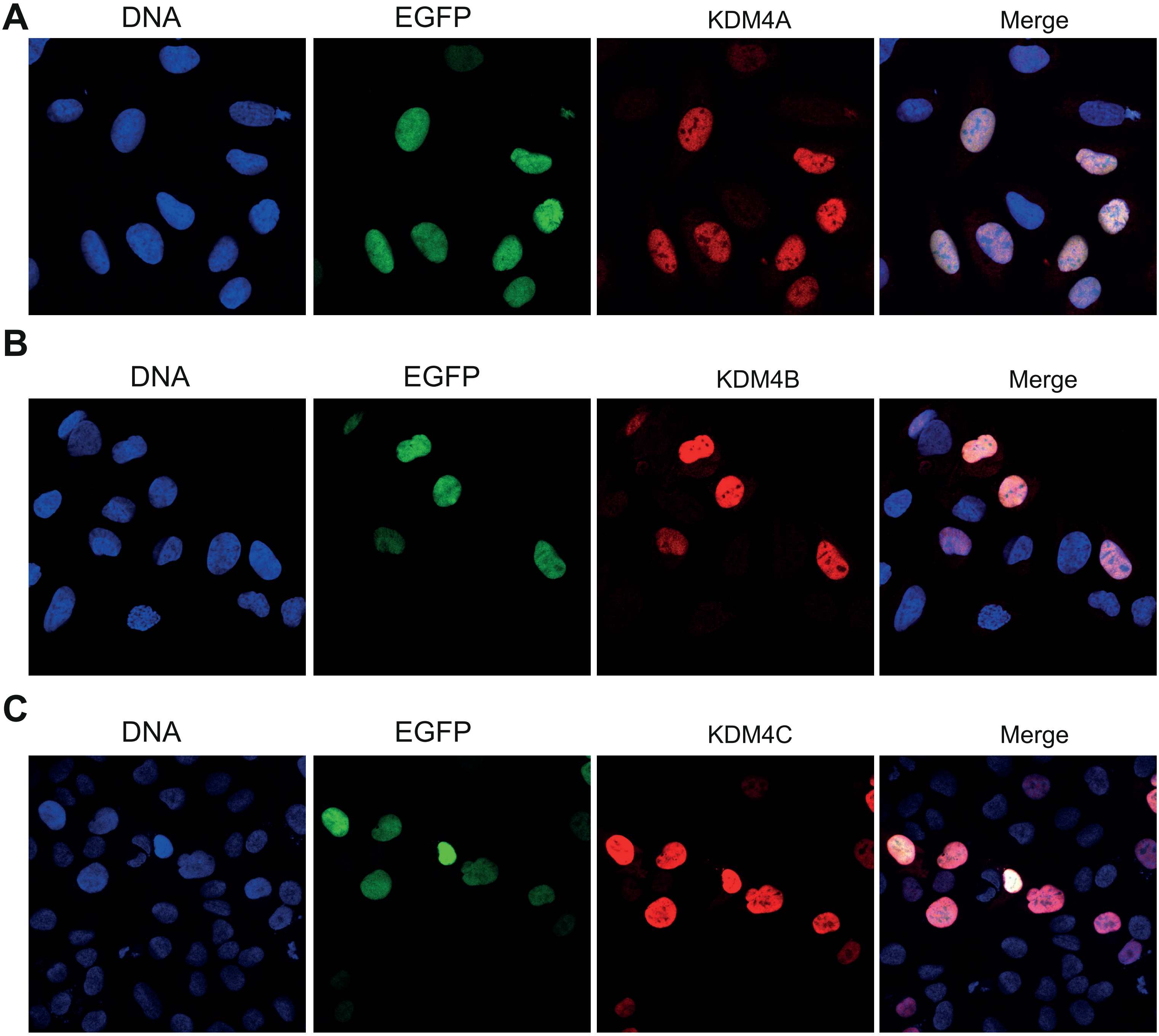

A
DNA
EGFP
KDM4A
Merge
B
DNA
EGFP
KDM4B
Merge
C
DNA
EGFP
KDM4C
Merge

Figure S1. Assessment of KDM4A-C antibodies suitability for immunofluorescence analysis. (A-C) Shows the specificity of KDM4A-C antibodies recognizes the native form of KDM4A-C proteins by immunofluorescence analysis. U2OS cells transfected with a vector expressing EGFP-KDM4A-C fusions were fixed and stained for KDM4A-C (red). DNA is stained with DAPI (blue), and the EGFP-KDM4A-C are in green. Results reveal that cells overexpressing EGFP-KDM4A-C show higher intensity of red signal compared to untransfected cells.
